# An Outline of Medical Chemistry, for the Use of Students

**Published:** 1855-06

**Authors:** 


ART. IV.—*An Outline of Medical Chemistry, for the Use of Students.* By B. Howard Rand, A. M., M. D., Professor of Chemistry in the Philadelphia College of Medicine, &c., &c. Philadelphia: Lindsay & Blakiston. 1855.

This is a duodecimo volume of some [illegible]60 pp., and from the casual glance we have given it, it seems to fulfil the promise of its title page and preface. It is not claimed to be a comprehensive work on chemistry, but it presents some peculiarities which will fit it for the use of a large class of students. It confines its attention to the narrower range of subjects included in a strictly medical chemistry. After a brief discussion of those general laws on which all chemical knowledge is based—the laws of attraction, combination, repulsion, light, heat, magnetism, &c.,—it proceeds at once to the study of those substances, found in the pharmacopœia; omitting mention of the history of metallurgy, the application of chemistry to the arts, and those other features of ordinary text-books not entirely pertaining to medical science. Physiological chemistry is also omitted, a feature which we look upon as a fault, even in a work of this scope.

The advantages of such a work are self-evident. The student of medicine aspires to be a chemist in only occasional instances. To devote that attention and labor necessary to a competent knowledge of analytical chemistry, would require more time than our limited curriculum allows, and take it from other branches of superior practical importance.

If it is possible to teach just so much chemistry as will enable the student to avoid incompatibilities of drugs, and to comprehend those ordinary manifestations of the chemistry of nature every day displayed, without entailing upon him the labor and research necessary to the true chemist, there is a great desideratum obtained. It is to meet this indication that this manual is published. We have our doubts about the result, but if it succeeds in divesting the science of that appearance of difficulty which deters so many students from doing it justice, we shall regard it as a great point gained.

